# Supplementary material for: Calcineurin-Dependent Homeostatic Response of C. elegans Muscle Cells upon Prolonged Activation of Acetylcholine Receptors
Source: Cells. 2023 Sep 3;12(17):2201. doi: 10.3390/cells12172201 (PMC10486475; doi:10.3390/cells12172201)
Supplement: Supplementary file 1 [file cells-12-02201-s001.zip › cells-2561366-supplementary.pdf]

Figure S1

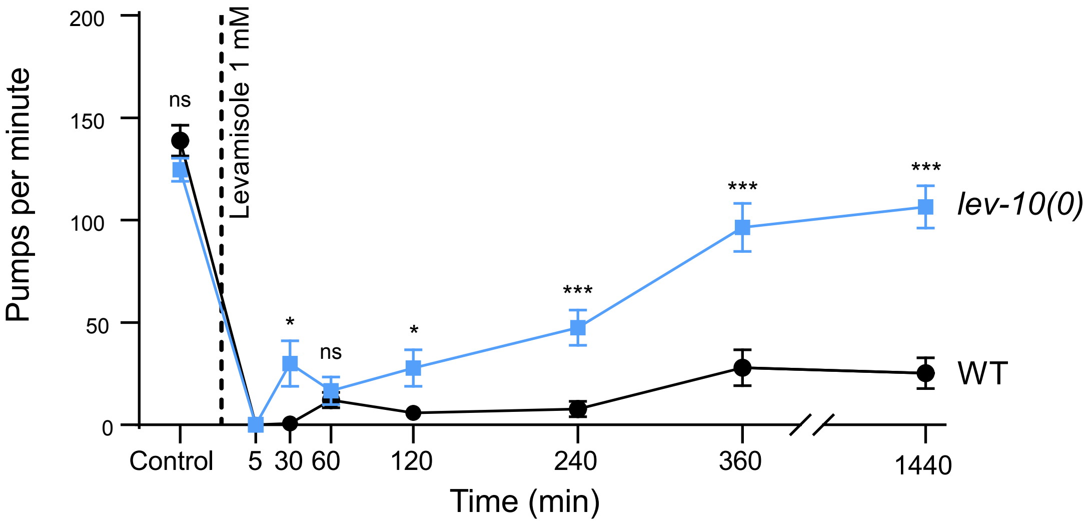

Pumps per minute
200
150
100
50
0
Levamisole 1 mM
ns
*
ns
*
***
***
***
lev-10(0)
WT
Control
5
30
60
120
240
360
1440
Time (min)

Figure S2

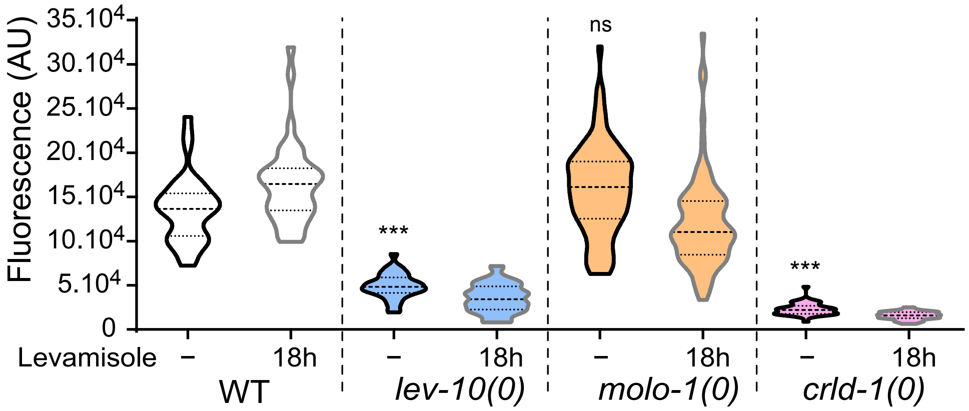
Fluorescence (AU)
35.10^4
30.10^4
25.10^4
20.10^4
15.10^4
10.10^4
5.10^4
0
ns
***
***
Levamisole
–
18h
–
18h
–
18h
–
18h
WT
lev-10(0)
molo-1(0)
crld-1(0)

Figure S3

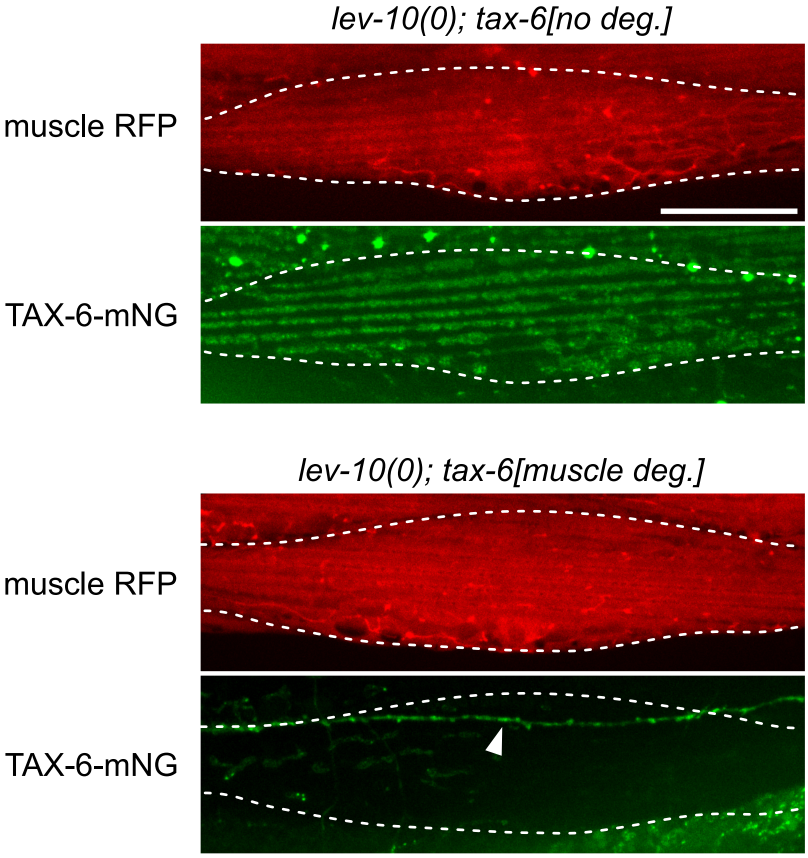
lev-10(0); tax-6[no deg.]
muscle RFP
TAX-6-mNG
lev-10(0); tax-6[muscle deg.]
muscle RFP
TAX-6-mNG

Figure S4

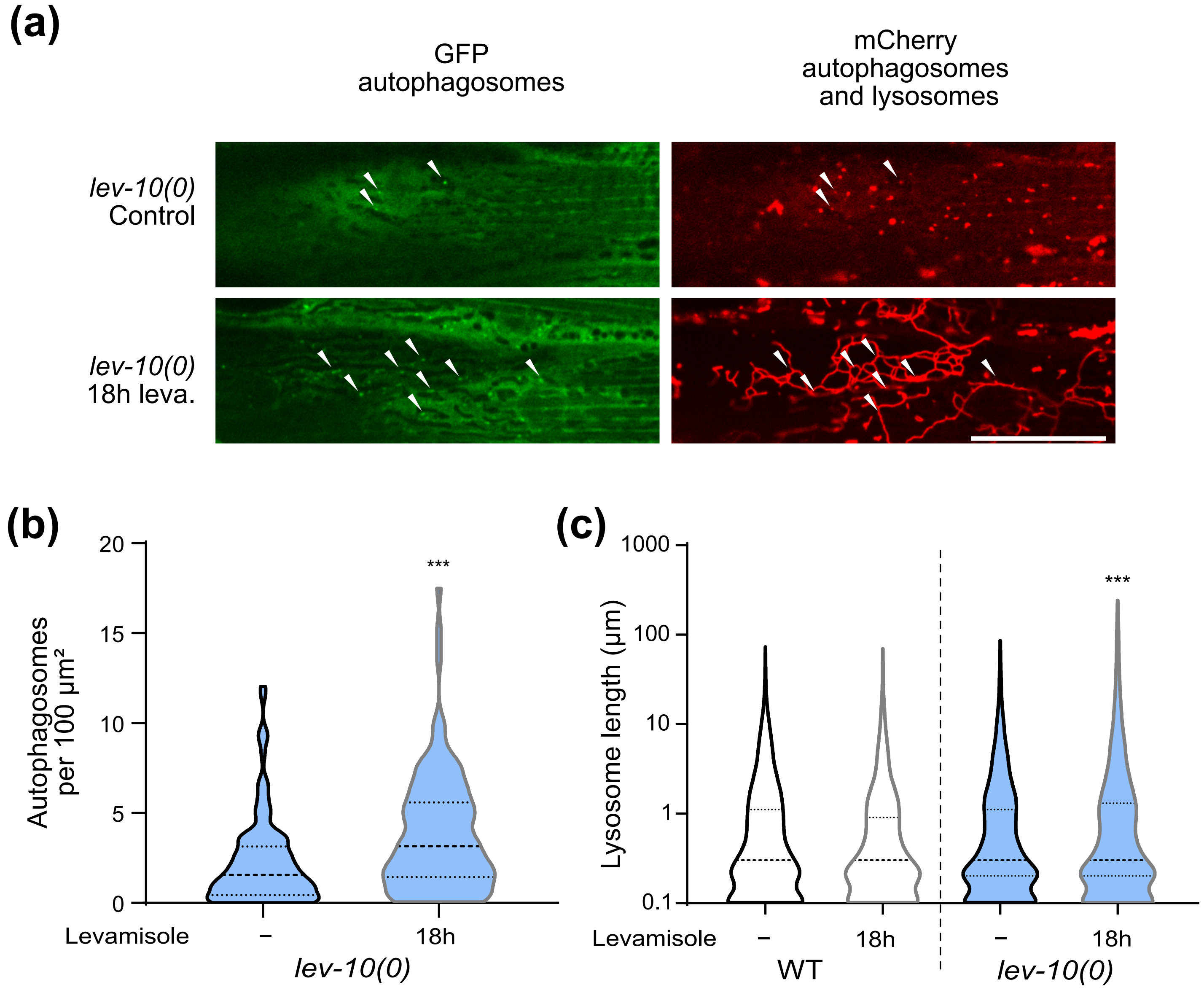
(a)
GFP
autophagosomes
mCherry
autophagosomes
and lysosomes
lev-10(0)
Control
lev-10(0)
18h leva.
(b)
20
15
10
5
0
Autophagosomes
per 100 µm²
***
Levamisole
–
18h
lev-10(0)
(c)
1000
100
10
1
0.1
Lysosome length (µm)
***
Levamisole
–
18h
–
18h
WT
lev-10(0)

Table S1. List of strains

Name	Genotype	Figure
N2	wild-type	Fig 1a, 1b, 1c, S1
EN26	*lev-10(kr26)I*	Fig 1a, 1b, 1c S1, 2c, 2d
EN8091	*molo-1(kr100)III*	Fig 1a, 1b
EN8269	*krTi3[Pmyo-3::GCaMP-6s-mCherry]V; lite-1(ce314)X*	Fig 1c
EN8270	*lev-10(kr26)I; krTi3[Pmyo-3::GCaMP-6s-mCherry]V; lite-1(ce314)X*	Fig 1c
EN8648	*molo-1(kr100)III; krTi3[Pmyo-3::GCaMP-6s-mCherry]V; lite-1(ce314)X*	Fig 1c
EN208	*unc-29(kr208[unc-29-TagRFP-T])I*	Fig 2a, 2b, S2a
EN1926	*unc-29(kr208[unc-29-TagRFP-T])I lev-10(kr26)I*	Fig 2a, 2b, S2a
EN5929	*unc-29(kr208[unc-29-TagRFP-T])I; crld-1(kr407)IV*	Fig 2a, 2b, S2a
EN7932	*lev-10(kr26)I; acr-16(kr440[acr-16-wScarlet])V*	Fig S2b
EN7925	*lev-10(kr26)I; unc-49(kr296[unc-49-RFP])III*	Fig S2c
CB1072	*unc-29(e1072am)I*	Table 1
EN8093	*molo-1(kr100)III; tax-6(kr423[tax-6-aid-mNG])IV; krSi55[pmyo-3::TIR1-eBFP]V*	Table 1
EN8649	*lev-10(kr26)I; aak-1(tm1944)III; aak-2(ok524)X*	Table 1
EN8152	*lev-10(kr26)I; cmk-1(ok287)IV*	Table 1
EN7391	*lev-10(kr26)I; crh-1(n3315)III*	Table 1
EN8241	*daf-16(mu86)I lev-10(kr26)I*	Table 1
EN7572	*lev-10(kr26)I; fzo-1(tm1133)II*	Table 1
EN8647	*lev-10(kr26)I; lgg-1(pp141)II*	Table 1
EN7267	*lev-10(kr26)I; gar-3(gk305)V*	Table 1
EN3100	*lev-10(kr26)I; mca-3(ar493)IV*	Table 1
EN7273	*lev-10(kr26)I; mcu-1(ju1154)IV*	Table 1
EN7501	*lev-10(kr26)I; mef-2(gk633)I*	Table 1
EN3849	*lev-10(kr26)I; slo-1(cx29)V; slo-2(nf100)X*	Table 1
EN3095	*lev-10(kr26)I; tax-6(db60)IV*	Table 1
EN8596	*lev-10(kr26)I; unc-43(e408)IV; zwIs102[Prab-3::YFP-unc-43]*	Table 1
EN3098	*lev-10(kr26)I; unc-68(r1162)V*	Table 1
EN7328	*lev-10(kr26)I; tax-6(kr423[tax-6-aid-mNG])IV; krSi55[Pmyo-3::TIR1-eBFP]V*	Fig 3a, 3b
EN8092	*lev-10(kr26)I; crh-1(bab180[crh-1-aid])III; krSi55[pmyo-3::TIR1-eBFP]V*	Table 1
EN7688	*lev-10(kr26)I; crtc-1(kr450[crtc-1-aid-wscarlet])I; krSi55[pmyo-3::TIR1-eBFP]V*	Table 1

EN7330	*lev-10(kr26)I; tax-6(kr423[tax-6-aid-mNG])IV; krSi50[Peft-3::TIR1-eBFP]IV*	Fig 3a
EN7382	*lev-10(kr26)I; tax-6(kr423[tax-6-aid-mNG])IV; krSi36[Prab-3::TIR1-eBFP]V*	Fig 3a
EN7384	*lev-10(kr26)I; tax-6(kr423[tax-6-aid-mNG])IV; krSi63[Pdpy-7::TIR1-eBFP]*	Fig 3a
EN8415	*lev-10(kr26)I; tax-6(kr540[tax-6-aid-eBFP])IV; krSi55[Pmyo-3::TIR1-eBFP]V krTi3[Pmyo-3::GCaMP-6s-mCherry]V; lite-1(ce314)X*	Fig 3c
EN7507	*unc-29(kr208[unc-29-TagRFP-T])I lev-10(kr26)I; tax-6(kr423[tax-6-aid-mNG])IV; krSi55[Pmyo-3::TIR1-eBFP]V*	Fig 3d
EN7714	*krSi134[Pmyo3::tom-20N-wScarlet] I*	Fig 4a, 4b
EN7716	*lev-10(kr26)I krSi134[Pmyo3::tom-20N-wScarlet]I*	Fig 4a, 4b
GA2001	*wuIs305[Pmyo-3::Queen-2m]*	Fig 4c
EN8374	*lev-10(kr26)I; wuIs305[Pmyo-3::Queen-2m]*	Fig 4c
KAG238	*kagIs1[Pdyc-1::GFP-lgg-1]*	Fig 5b
EN8162	*lev-10(kr26)I; kagIs1[Pdyc-1::GFP-lgg-1]*	Fig 5a, 5b
EN8245	*lev-10(kr26)I; kagIs3[Pdyc-1::GFP-lgg-1G116A]*	Fig 5b
EN8238	*lev-10(kr26)I; tax-6(kr540[tax-6-aid-eBFP])IV; krSi55[Pmyo-3::TIR1-eBFP]V; kagIs1[Pdyc-1::GFP-lgg-1]*	Fig 6a
EN8363	*krSi114[Pmyo-3::lmp-1-eBFP]*	Fig 5d
EN8365	*lev-10(kr26)I; krSi114[Pmyo-3::lmp-1-eBFP]*	Fig 5c, d
EN8412	*lev-10(kr26)I; tax-6(kr423[tax-6-aid-mNG])IV; krSi55[Pmyo-3::TIR1-eBFP] V; krSi114[Pmyo-3::lmp-1-eBFP]*	Fig 6b
EN8961	*lev-10(kr26)I krSi134[Pmyo3::tom-20N-wScarlet]I; tax-6(kr540[tax-6-aid-eBFP)IV; krSi55[Pmyo-3::TIR1-eBFP]V*	Fig 6c
EN7380	*lev-10(kr26::Mos1)I; tax-6(kr423[tax-6-aid-mNG])IV; krSi55[Pmyo-3::TIR1-eBFP]V; tnt-2(fs410[tnt-2::SL2::tagRFP-T])X*	Fig S3a
EN8236	*lev-10(kr26)I; sqIs11[Plgg-1::mCherry-GFP-lgg-1]*	Fig S4
